# Supplementary material for: Global determinants of insect mitochondrial genetic diversity
Source: Nat Commun. 2023 Aug 29;14:5276. doi: 10.1038/s41467-023-40936-0 (PMC10465557; doi:10.1038/s41467-023-40936-0)
Supplement: Supplementary file 1 — Supplementary Information [file 41467_2023_40936_MOESM1_ESM.pdf]

Supplementary Information

Global distribution of insect mitochondrial genetic diversity

French et al.

Supplementary Fig. 1

The observed distribution of genetic diversity mean (GDM) (a, c, e, g, i, k) and genetic diversity evenness (GDE) (b, d, f, h, l) for datasets with a minimum operational taxonomic unit (OTU) per-cell threshold of 10 (a, b), 25 (c, d), 50 (e, f), 100 (g, h), 150 (i, j), and 200 (k, l). The number of sampled cells is 957, 572, 376, 245, 189, and 160, respectively for each OTU threshold.

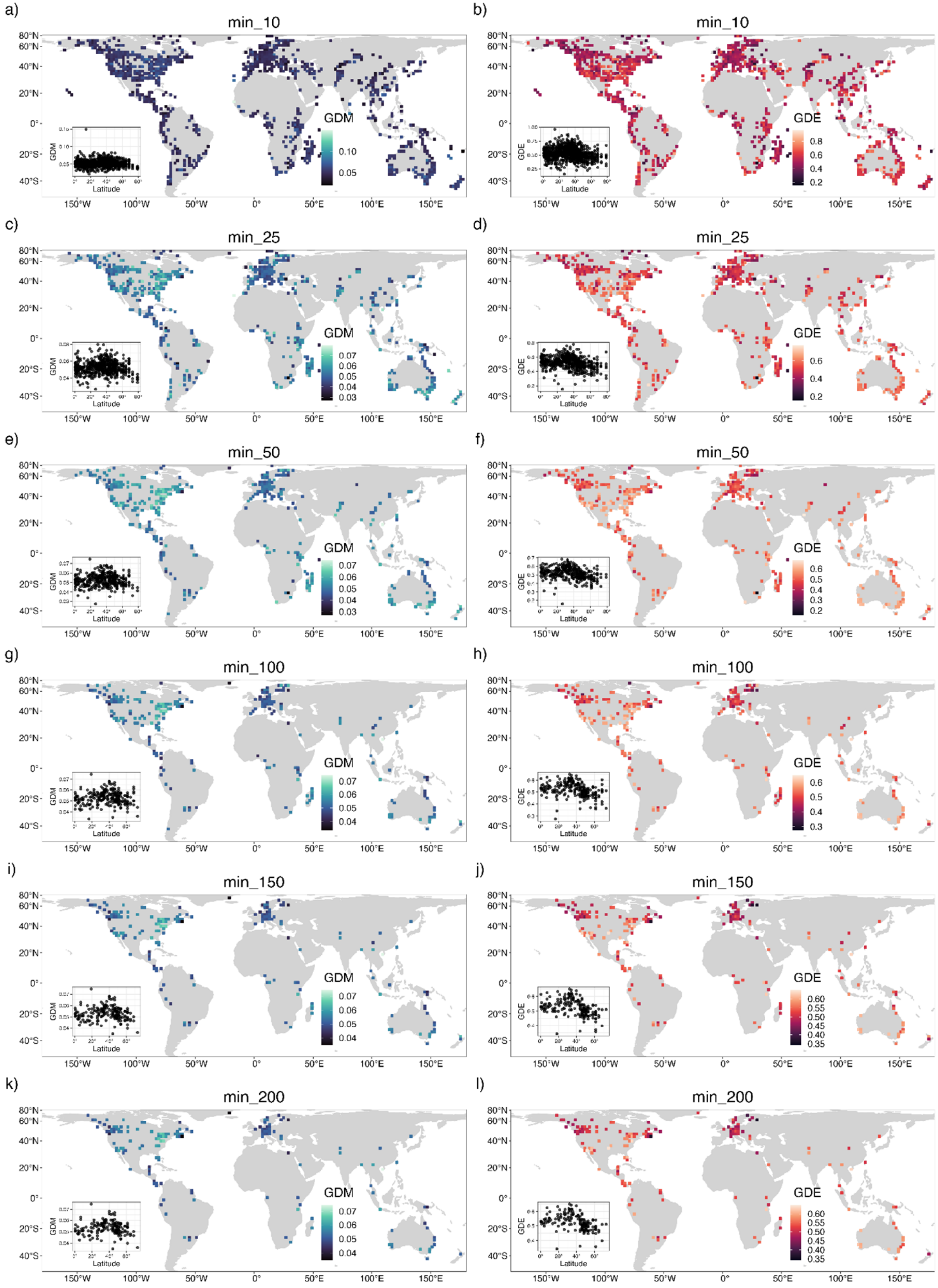

a)
min_10
GDM
0.10
0.05
b)
min_10
GDE
0.8
0.6
0.4
0.2
c)
min_25
GDM
0.07
0.06
0.05
0.04
0.03
d)
min_25
GDE
0.6
0.4
0.2
e)
min_50
GDM
0.07
0.06
0.05
0.04
0.03
f)
min_50
GDE
0.6
0.5
0.4
0.3
0.2
g)
min_100
GDM
0.07
0.06
0.05
0.04
h)
min_100
GDE
0.6
0.5
0.4
0.3
i)
min_150
GDM
0.07
0.06
0.05
0.04
j)
min_150
GDE
0.60
0.55
0.50
0.45
0.40
0.35
k)
min_200
GDM
0.07
0.06
0.05
0.04
l)
min_200
GDE
0.60
0.55
0.50
0.45
0.40
0.35
80°N
60°N
40°N
20°N
0°
20°S
40°S
150°W
100°W
50°W
0°
50°E
100°E
150°E
Latitude

Supplementary Fig. 2

Criteria used to select the grid cell resolution and minimum number of operational taxonomic units (OTUs) per cell for this study. The three resolutions considered were 96.5 km x 96.5 km ("high"), 193 km x 193 km ("medium"), and 385.9 km x 385.9 km ("low") equal-area grid cells using a Behrmann cylindrical equal-area projection. Each plot has the spatial resolution along the x-axis and the total number of cells along the y-axis. We additionally screened for a sampling scheme that balances the number of grid cells (a), median number of orders (b),

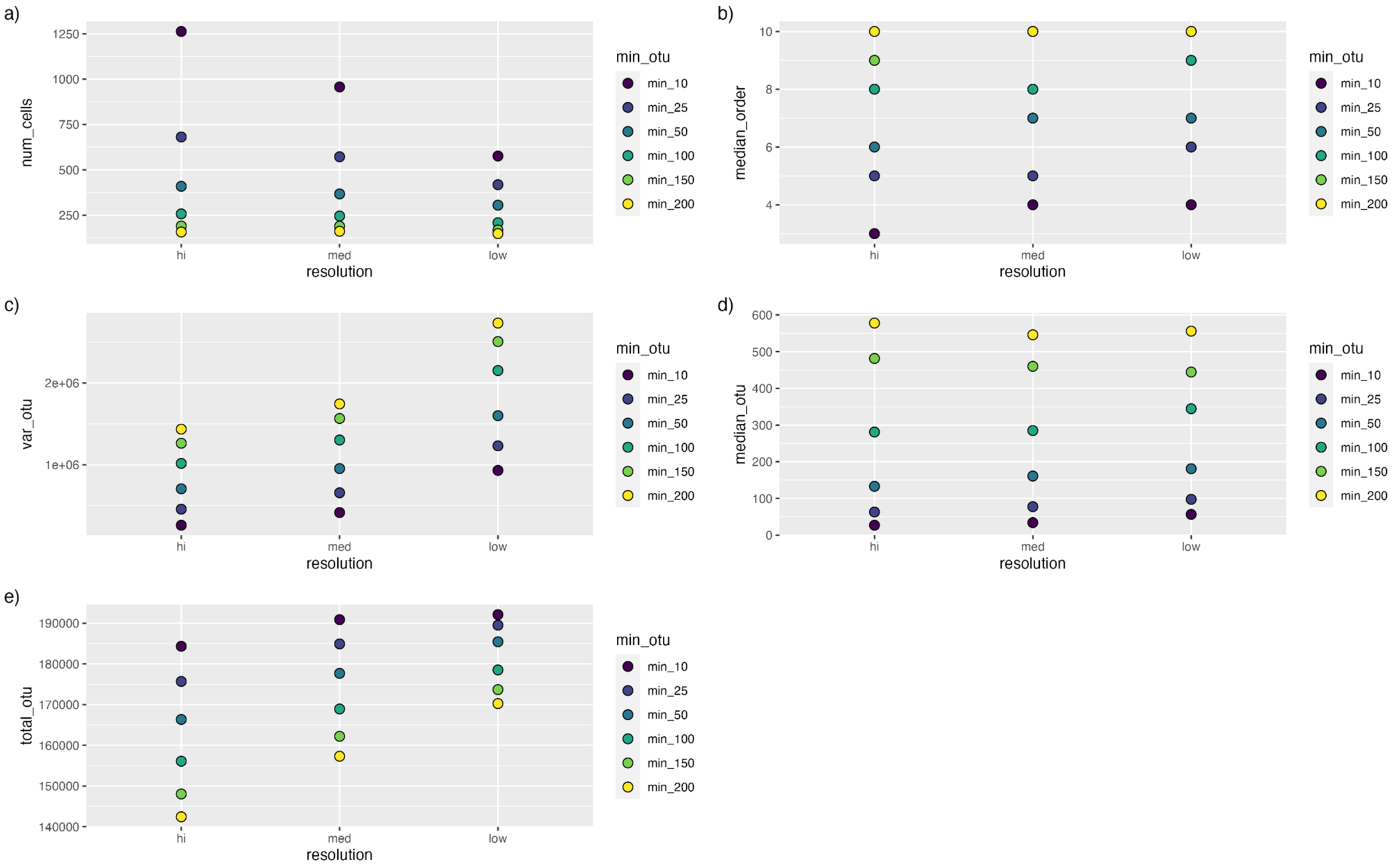

variation in the number of OTUs across cells (c), median number of orders per cell (d), and total number of OTUs (e).

Supplementary Fig. 3

Observed data versus predicted data from spatial generalized linear mixed models trained on 75% of data with 25% of the data withheld. Black lines indicate a perfect 1-to-1 relationship between observed and predicted data, and red lines with gray shading are ordinary least squares trend lines with 95% confidence intervals from a regression between observed and predicted data. Points are colored according to the continent they were sampled in, demonstrating little spatial bias in residuals. Below each plot are summary statistics indicating model fit, where RMSE is the root mean squared error and Y-int is the y-intercept. Lower RMSE and higher R^2 values indicate strong explanatory power, while a slope near one and a y-intercept near zero indicate low prediction bias.

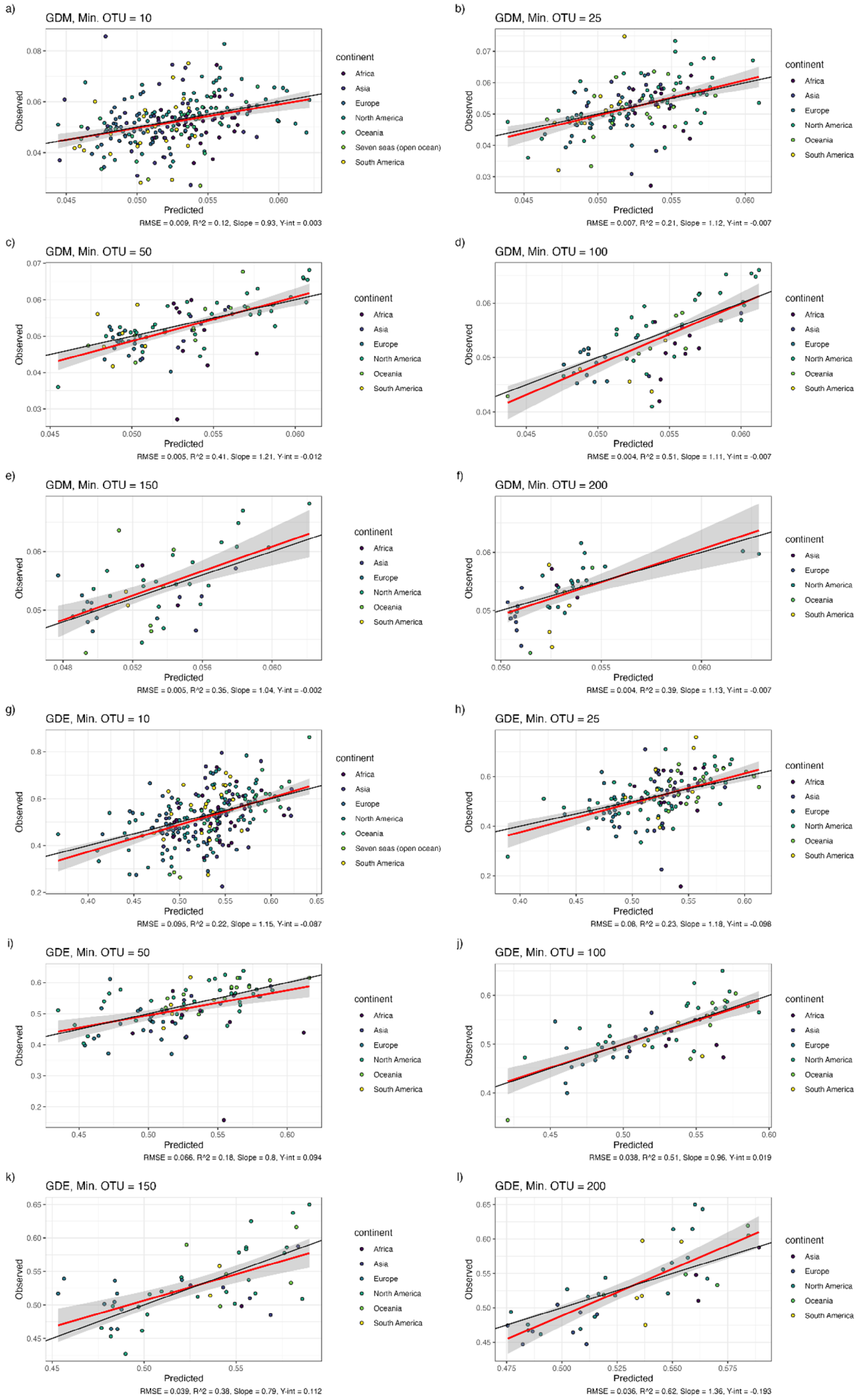
a)
GDM, Min. OTU = 10
Observed
Predicted
RMSE = 0.009, R^2 = 0.12, Slope = 0.93, Y-int = 0.003
continent
Africa
Asia
Europe
North America
Oceania
Seven seas (open ocean)
South America
b)
GDM, Min. OTU = 25
RMSE = 0.007, R^2 = 0.21, Slope = 1.12, Y-int = -0.007
c)
GDM, Min. OTU = 50
RMSE = 0.005, R^2 = 0.41, Slope = 1.21, Y-int = -0.012
d)
GDM, Min. OTU = 100
RMSE = 0.004, R^2 = 0.51, Slope = 1.11, Y-int = -0.007
e)
GDM, Min. OTU = 150
RMSE = 0.005, R^2 = 0.35, Slope = 1.04, Y-int = -0.002
f)
GDM, Min. OTU = 200
RMSE = 0.004, R^2 = 0.39, Slope = 1.13, Y-int = -0.007
g)
GDE, Min. OTU = 10
RMSE = 0.095, R^2 = 0.22, Slope = 1.15, Y-int = -0.087
h)
GDE, Min. OTU = 25
RMSE = 0.08, R^2 = 0.23, Slope = 1.18, Y-int = -0.098
i)
GDE, Min. OTU = 50
RMSE = 0.066, R^2 = 0.18, Slope = 0.8, Y-int = 0.094
j)
GDE, Min. OTU = 100
RMSE = 0.038, R^2 = 0.51, Slope = 0.96, Y-int = 0.019
k)
GDE, Min. OTU = 150
RMSE = 0.039, R^2 = 0.38, Slope = 0.79, Y-int = 0.112
l)
GDE, Min. OTU = 200
RMSE = 0.036, R^2 = 0.62, Slope = 1.36, Y-int = -0.193

Supplementary Fig. 4

Global hotspots and coldspots of genetic diversity. These maps indicate grid cells in the top (red) and bottom (blue) 10% quantile of GDM and together (a-d), GDM alone (e-h), and GDE alone (i-l). Panels on the left are projections of the summary statistics across the globe from a spatial Bayesian GLMM model using environmental predictors (see Results) and panels on the right depict observed data. In the left-hand panels, areas with environments non-analogous to the environments used for modeling were removed prior to calculating the 10% quantiles.

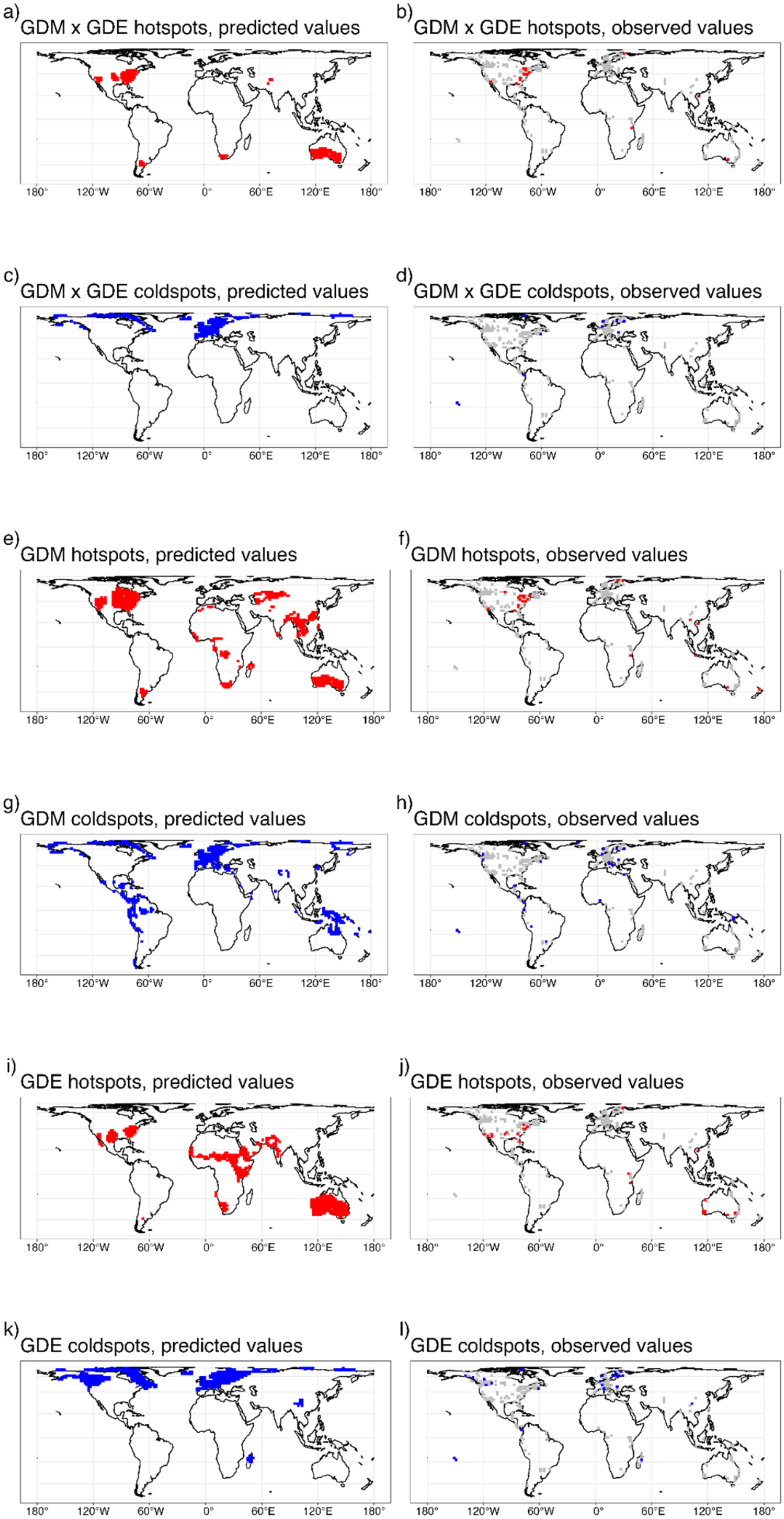
a) GDM x GDE hotspots, predicted values
b) GDM x GDE hotspots, observed values
c) GDM x GDE coldspots, predicted values
d) GDM x GDE coldspots, observed values
e) GDM hotspots, predicted values
f) GDM hotspots, observed values
g) GDM coldspots, predicted values
h) GDM coldspots, observed values
i) GDE hotspots, predicted values
j) GDE hotspots, observed values
k) GDE coldspots, predicted values
l) GDE coldspots, observed values
180° 120°W 60°W 0° 60°E 120°E 180°

Supplementary Fig. 5

Global maps of the top environmental predictors of GDM and GDE. MTWM is the maximum temperature of the warmest month bioclimatic variable (°C * 10) (a), Temp. Trend describes centennial trends in temperature variation since the last glacial maximum (LGM) (b), PWM is the precipitation of the wettest month bioclimatic variable (mm) (c), Precip. Trend describes centennial trends in precipitation variation since the LGM (d), Temp. Range is the mean diurnal air temperature range bioclimatic variable (ºC * 10) (e), Precip. Seasonality is the precipitation seasonality bioclimatic variable (kg m^{-2}) (f), Precip. Variation is the standard deviation around centennial trends in precipitation variation since the LGM (g), and PDM is the precipitation of the driest month bioclimatic variation (kg m^{-2}) (h). Precip. Seasonality, PWM, PDM, Temp. Trend, Precip. Variation, MTWM, Temp. Range, and Precip. Trend were the eight predictors in a Bayesian GLMM model of GDM, while Temp. Trend, PWM, and MTWM were the three predictors in a model of GDM.

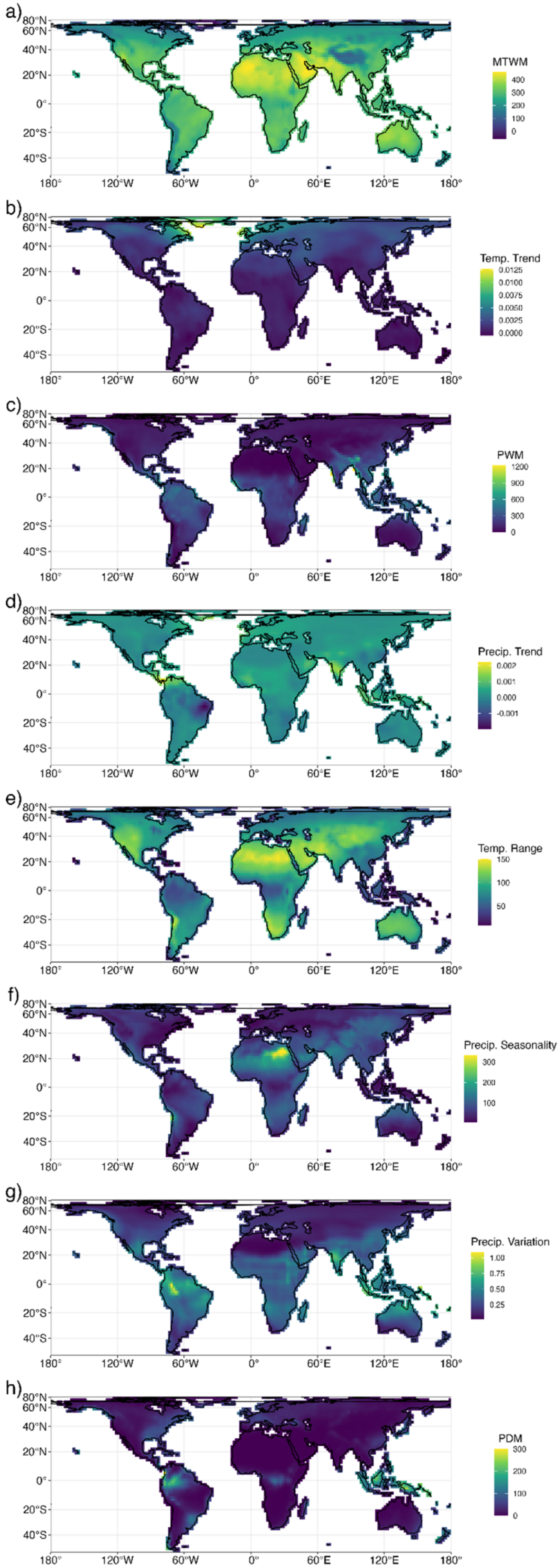
a)
80°N
60°N
40°N
20°N
0°
20°S
40°S
180°
120°W
60°W
0°
60°E
120°E
180°
MTWM
400
300
200
100
0
b)
Temp. Trend
0.0125
0.0100
0.0075
0.0050
0.0025
0.0000
c)
PWM
1200
900
600
300
0
d)
Precip. Trend
0.002
0.001
0.000
-0.001
e)
Temp. Range
150
100
50
f)
Precip. Seasonality
300
200
100
g)
Precip. Variation
1.00
0.75
0.50
0.25
h)
PDM
300
200
100
0

Supplementary Fig. 6

The residuals of genetic diversity mean (GDM) (a) and genetic diversity evenness (GDE) (b) for the reported dataset with a minimum operational taxonomic unit (OTU) threshold of 100. The residuals had no spatial autocorrelation (see Table 2).

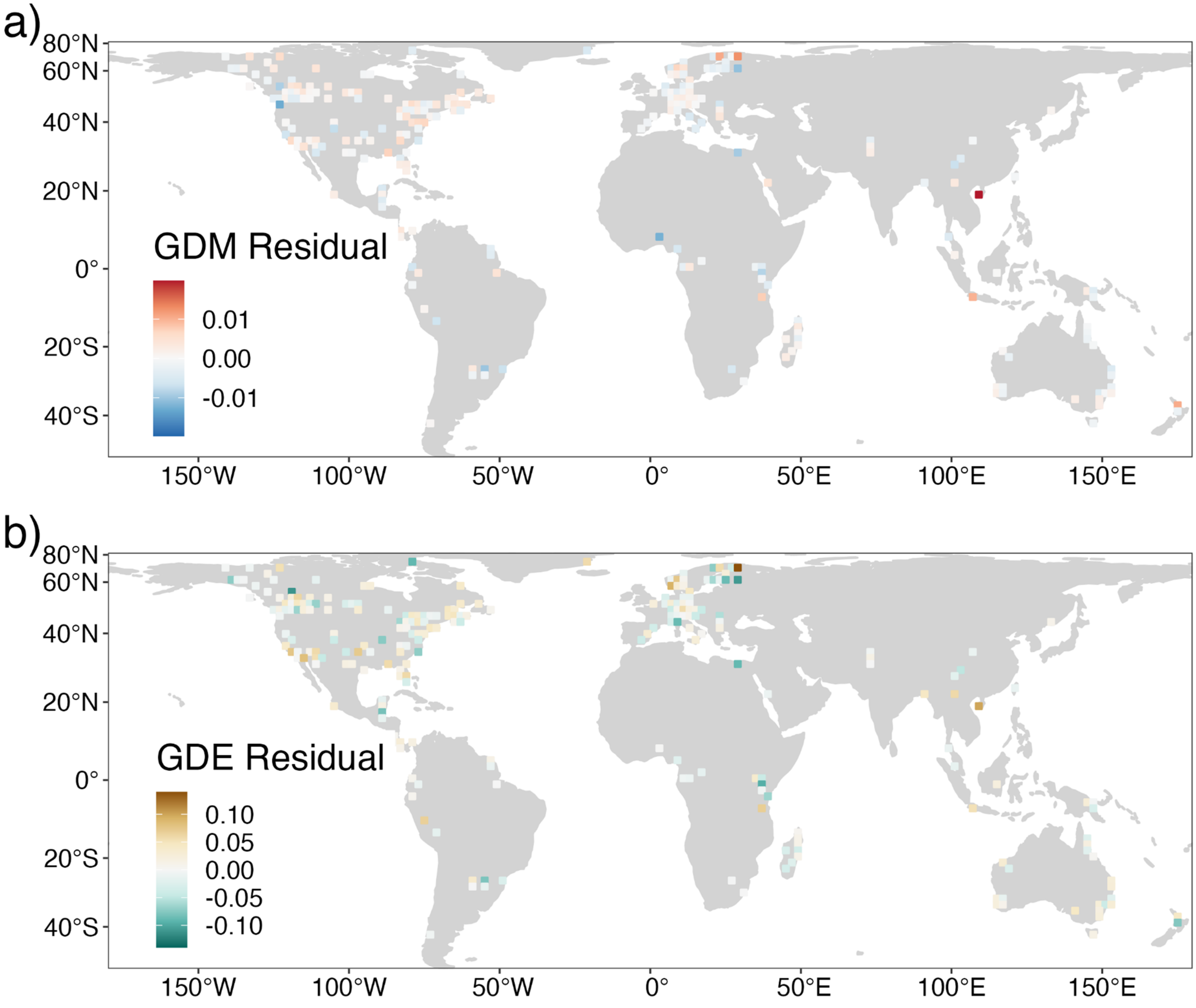

Supplementary Fig. 7

Trace plots and prior-posterior overlap plots indicate model stability and parameter identifiability respectively for genetic diversity mean (GDM) (a) and genetic diversity evenness (GDE) (b). The variables considered for GDM (a) are the intercept (B[1]), precipitation trend (B[2]), temperature range (B[3]), maximum temperature of the warmest month (B[4]), precipitation seasonality (B[5]), temperature trend (B[6]), precipitation variation (B[7]), precipitation of the wettest month (B[8]), precipitation of the driest month (B[9]) . The variables considered for GDE (b) are the intercept (B[1]), maximum temperature of the warmest month (B[2]), temperature trend (B[3]), and precipitation of the wettest month (B[4]). Percentages indicate the percentage of the prior distribution that the posterior distribution overlaps with.

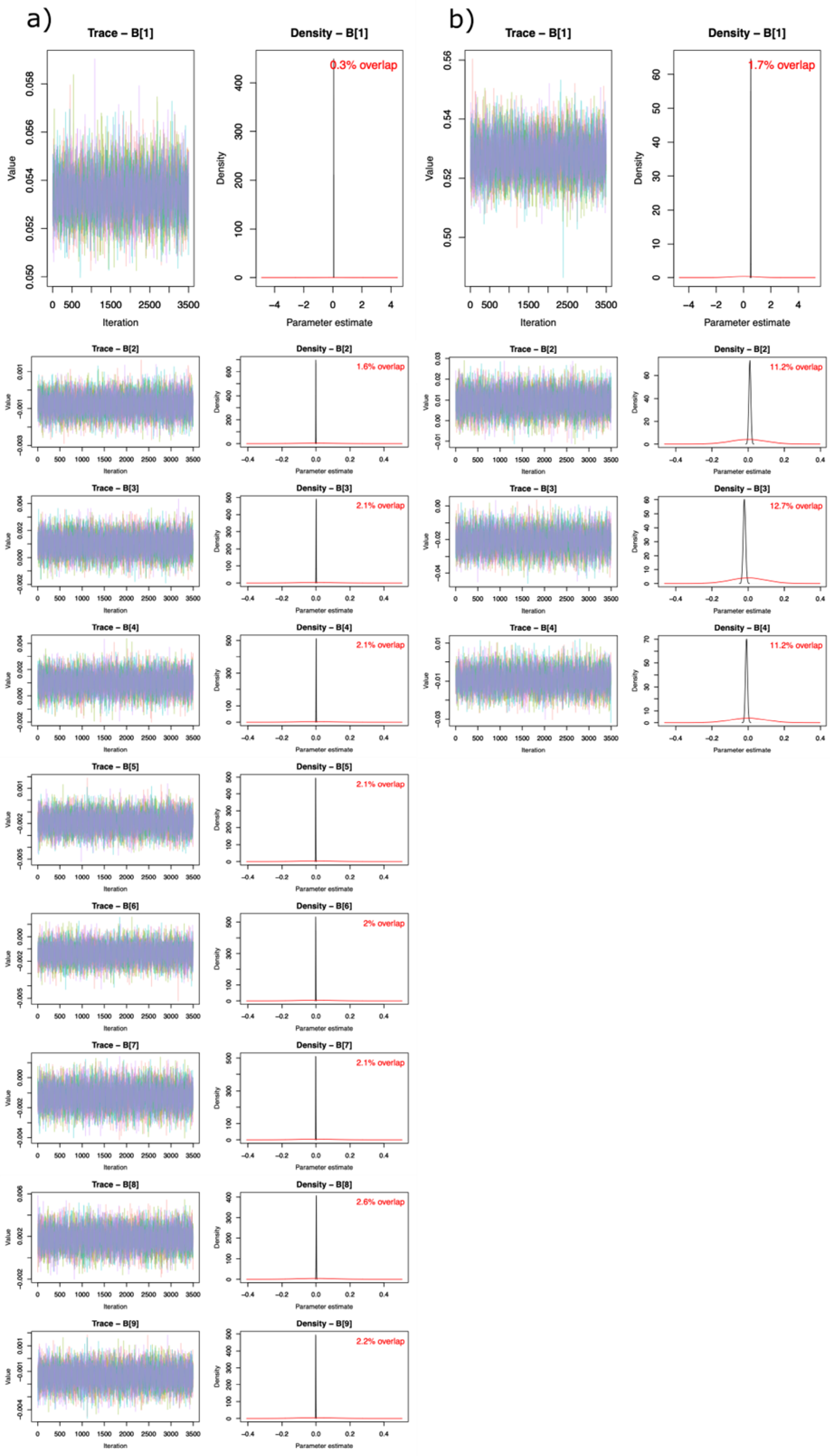
a)
Trace – B[1]
Density – B[1]
0.3% overlap
Trace – B[2]
Density – B[2]
1.6% overlap
Trace – B[3]
Density – B[3]
2.1% overlap
Trace – B[4]
Density – B[4]
2.1% overlap
Trace – B[5]
Density – B[5]
2.1% overlap
Trace – B[6]
Density – B[6]
2% overlap
Trace – B[7]
Density – B[7]
2.1% overlap
Trace – B[8]
Density – B[8]
2.6% overlap
Trace – B[9]
Density – B[9]
2.2% overlap
b)
Trace – B[1]
Density – B[1]
1.7% overlap
Trace – B[2]
Density – B[2]
11.2% overlap
Trace – B[3]
Density – B[3]
12.7% overlap
Trace – B[4]
Density – B[4]
11.2% overlap
Value
Iteration
Density
Parameter estimate

Supplementary Fig. 8

Maps showing the global distribution of model uncertainty in genetic diversity mean (GDM) (a, b, c) and genetic diversity evenness (GDE) (d, e, f). The upper 95% highest density interval (HDI) (a, d) and lower 95% HDI (b, e) are plotted. The upper 95% HDI and lower 95% HDI broadly reflect similar patterns to the reported median value (see Fig. 2). We masked in gray areas with environments non-analogous to the environments used for modeling.

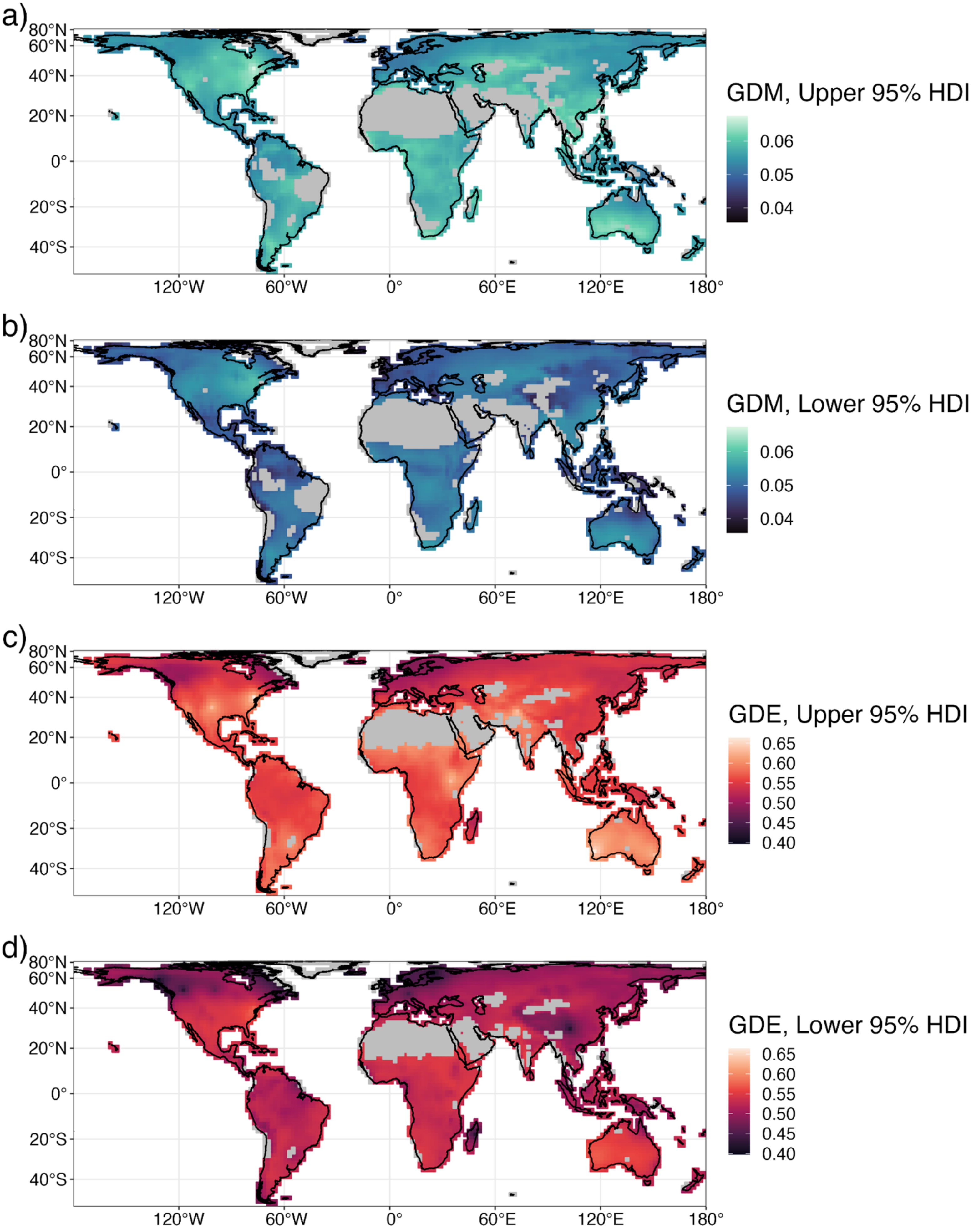

a)
GDM, Upper 95% HDI
0.06
0.05
0.04
b)
GDM, Lower 95% HDI
0.06
0.05
0.04
c)
GDE, Upper 95% HDI
0.65
0.60
0.55
0.50
0.45
0.40
d)
GDE, Lower 95% HDI
0.65
0.60
0.55
0.50
0.45
0.40
80°N
60°N
40°N
20°N
0°
20°S
40°S
120°W
60°W
0°
60°E
120°E
180°

Supplementary Fig. 9

Multivariate environmental similarity surfaces, which indicate environmental space that is analogous and non-analogous to that used to train the spatial Bayesian generalized linear mixed models of genetic diversity mean (GDM) (a) and genetic diversity evenness (GDE) (b). We masked areas with non-analogous climate in our final projections to limit model extrapolation and uncertainty.

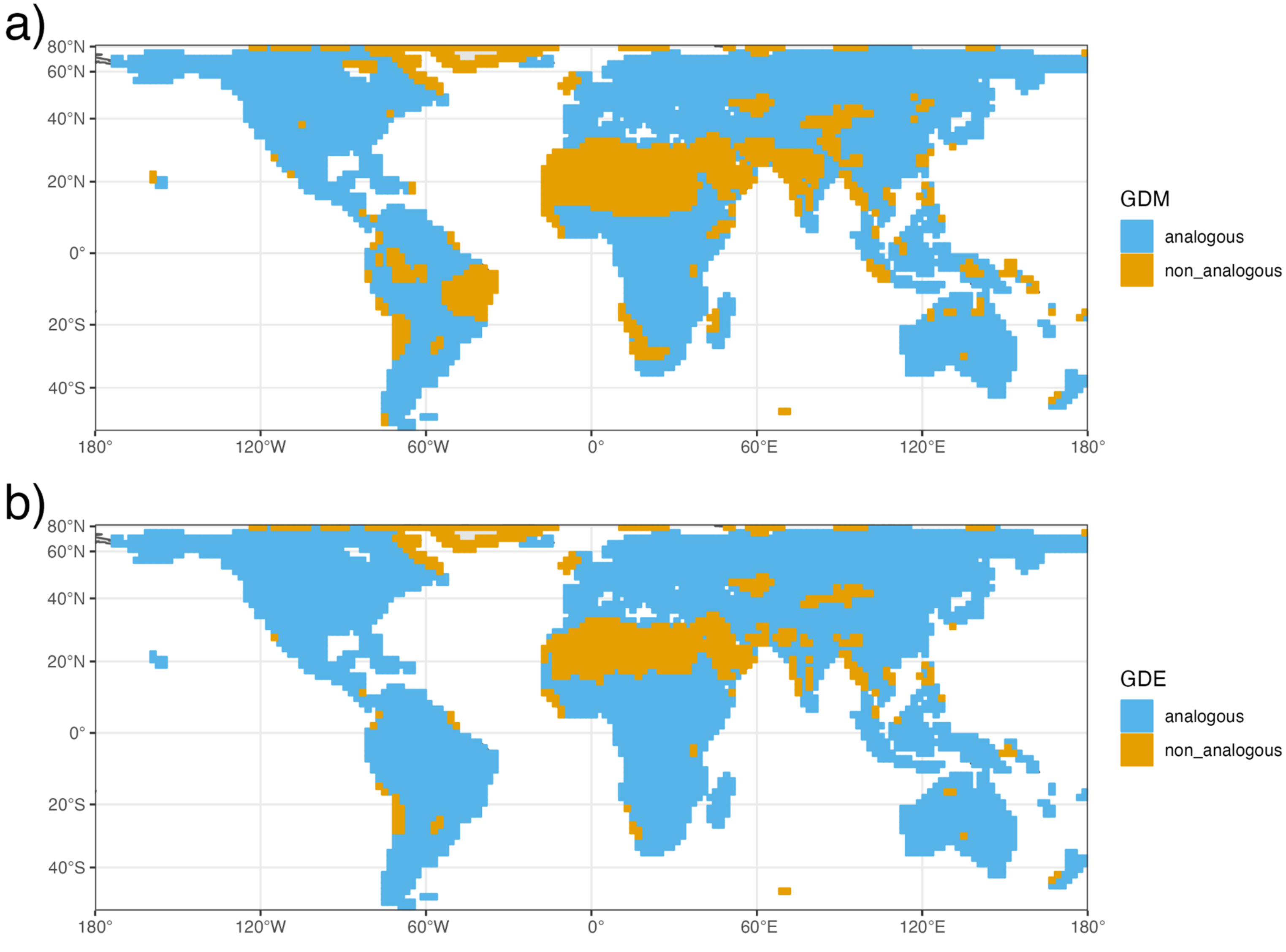
a)
80°N
60°N
40°N
20°N
0°
20°S
40°S
180°
120°W
60°W
0°
60°E
120°E
180°
GDM
analogous
non_analogous
b)
80°N
60°N
40°N
20°N
0°
20°S
40°S
180°
120°W
60°W
0°
60°E
120°E
180°
GDE
analogous
non_analogous

Supplementary Fig. 10

The proportion of operational taxonomic units (OTUs) belonging to each insect order from the observed data set. Three orders contain over 10% of sampled OTUs each, while 20 orders contain fewer than 5% of sampled OTUs in total (“Other”) (see Supplementary Table 3 for full

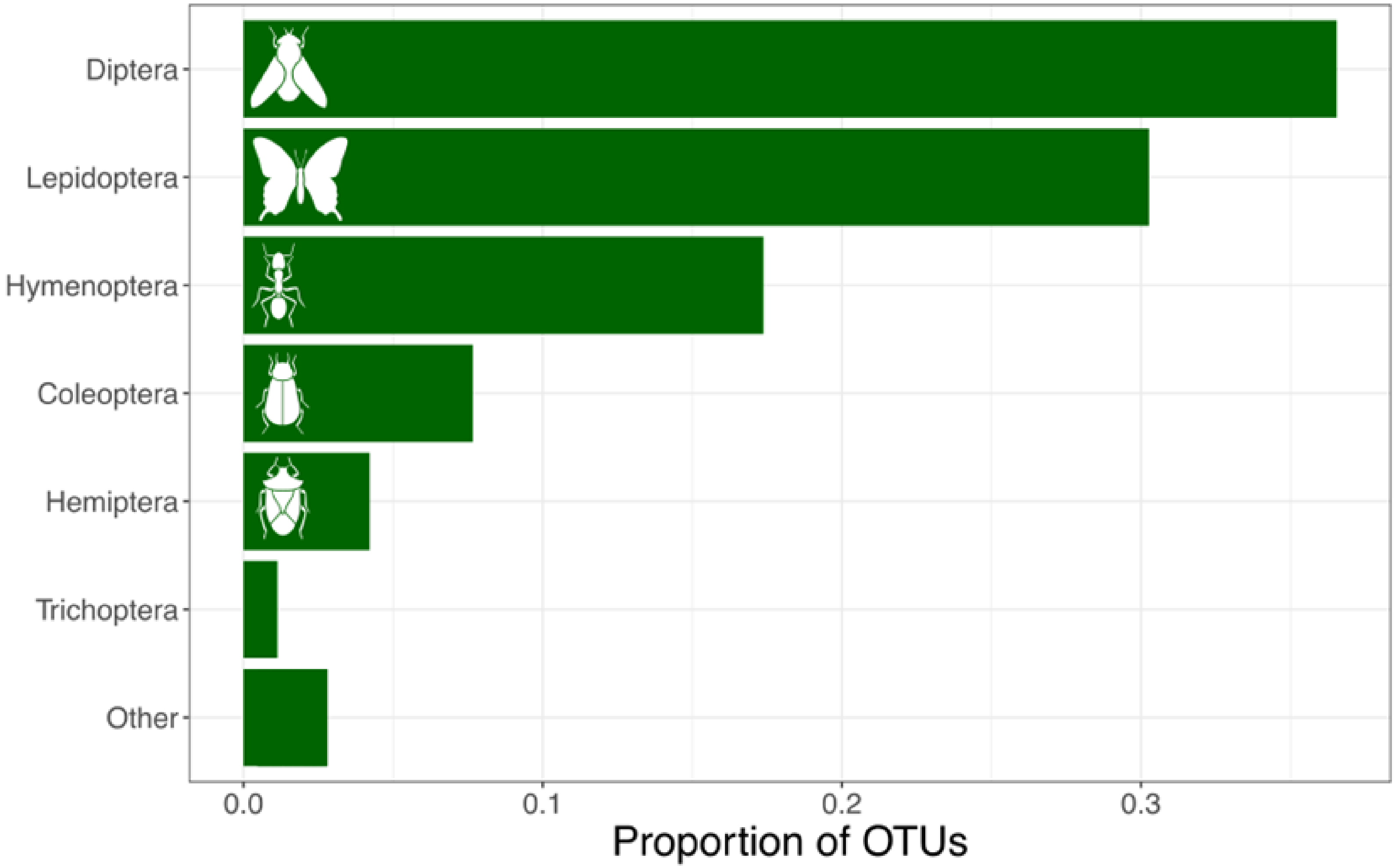

sampling information).

Supplementary Fig. 11

The distribution of the number of grid cells each operational taxonomic unit (OTU) occupies. The tick marks along the x-axis indicate the presence of OTUs that occupy 25 or more cells, but are not visible on the histogram. Over 74 percent of OTUs occupy a single grid cell and over 99 percent occupy fewer than 12 grid cells.

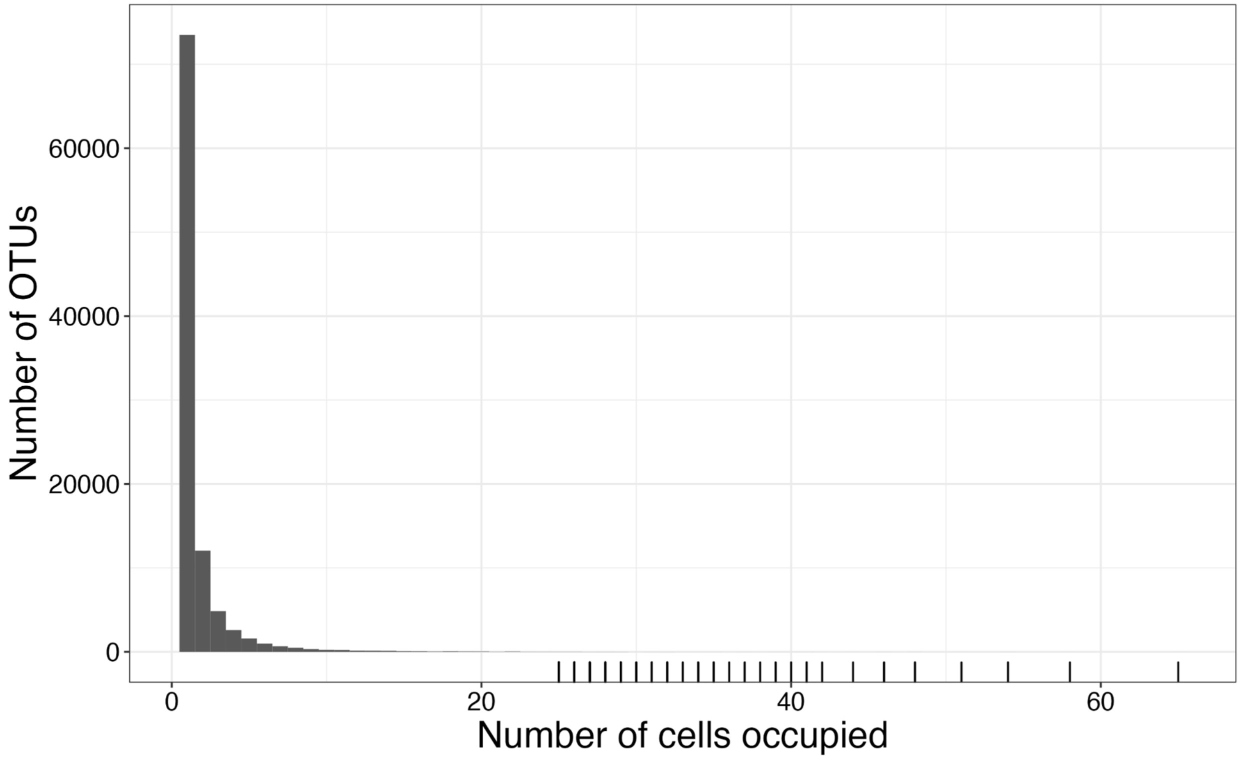

Supplementary Fig. 12

The per-cell distributions of genetic diversity mean (GDM) (a) and genetic diversity evenness (GDE) (b) with the most-sampled orders removed compared to the full data set. The boxplot center represents the median of the data, while the lower and upper hinges correspond to the first and third quartiles. The whiskers extend to the largest value no further than 1.5 times the inter-quartile range from the hinge. Three orders included over 10% of sampled operational taxonomic units (OTUs) – Diptera (34.0%), Lepidoptera (32.4%), and Hymenoptera (17.3%) (see Supplementary Table 3 for full sampling information). The two datasets with the top three orders removed contained N = 82 cells.

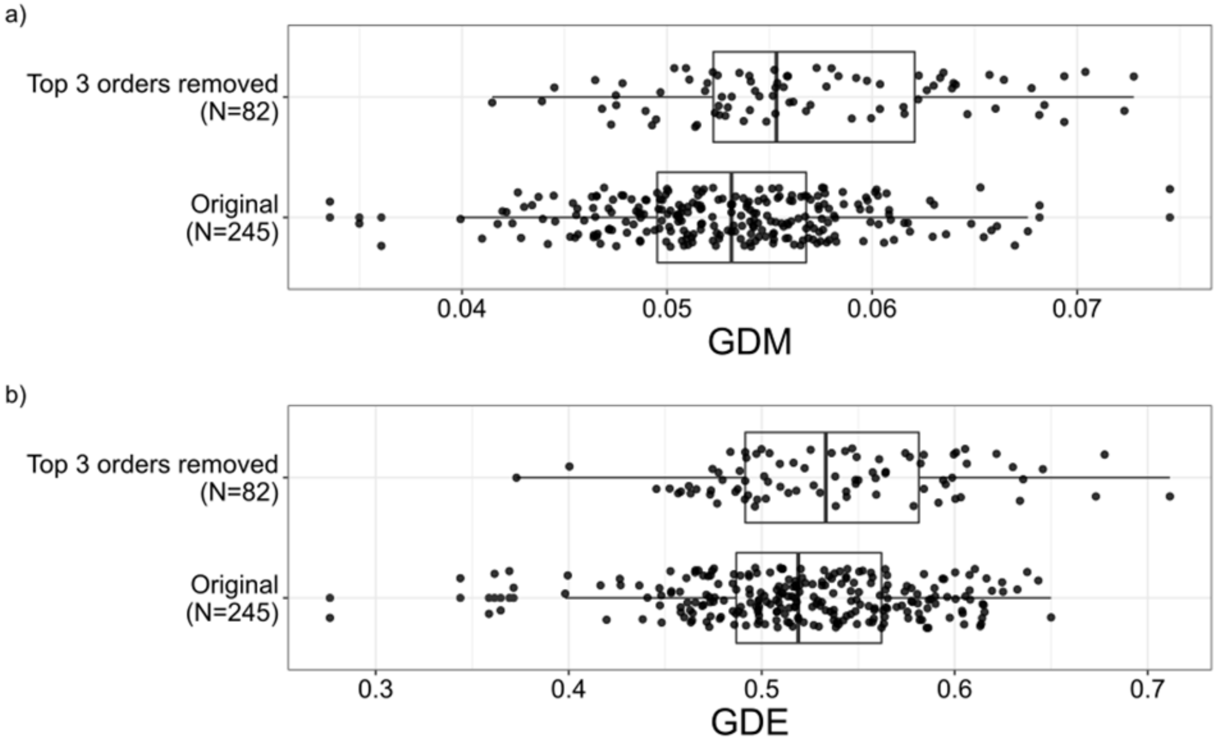

Supplementary Fig. 13

The taxonomic distribution of operational taxonomic unit (OTU) sampling varied geographically. Of the three most sampled orders, Diptera (a) dominated sampling towards the poles and Lepidoptera (b) dominated sampling in the tropics and in some temperate localities at middle latitudes. Hymenoptera (c) typically consisted of fewer than 50% of OTUs sampled per grid cell, although it dominated sampling in Madagascar. Proportions were derived from the number of OTUs sampled per cell belonging to each order.

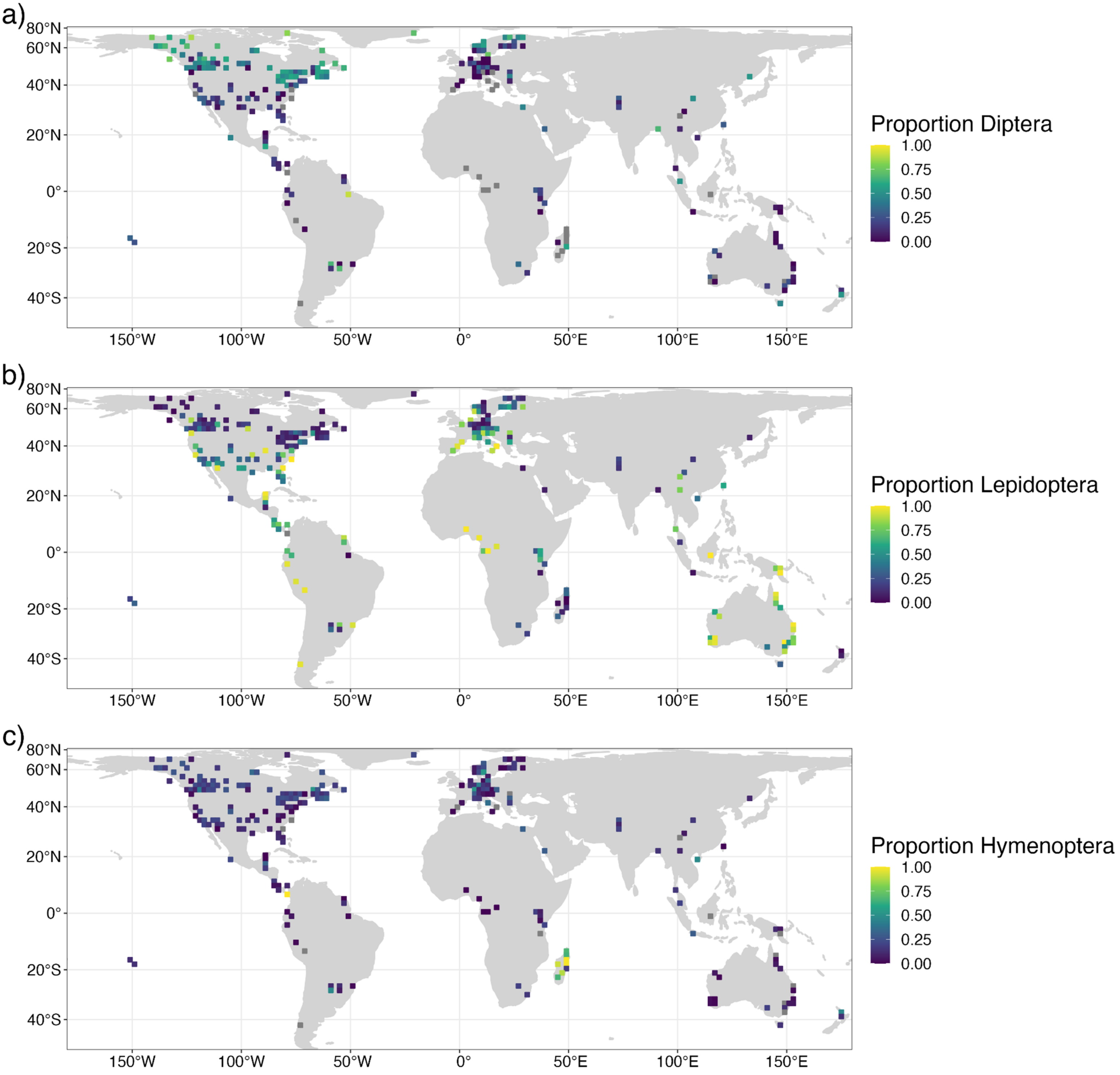
a)
b)
c)
80°N
60°N
40°N
20°N
0°
20°S
40°S
150°W
100°W
50°W
0°
50°E
100°E
150°E
Proportion Diptera
Proportion Lepidoptera
Proportion Hymenoptera
1.00
0.75
0.50
0.25
0.00

Supplementary Fig. 14

The change in the number of grid cells occupied per operational taxonomic unit (OTU) with latitude. The OTUs are binned, showing the vast majority of OTUs in the data set occupy few grid cells.

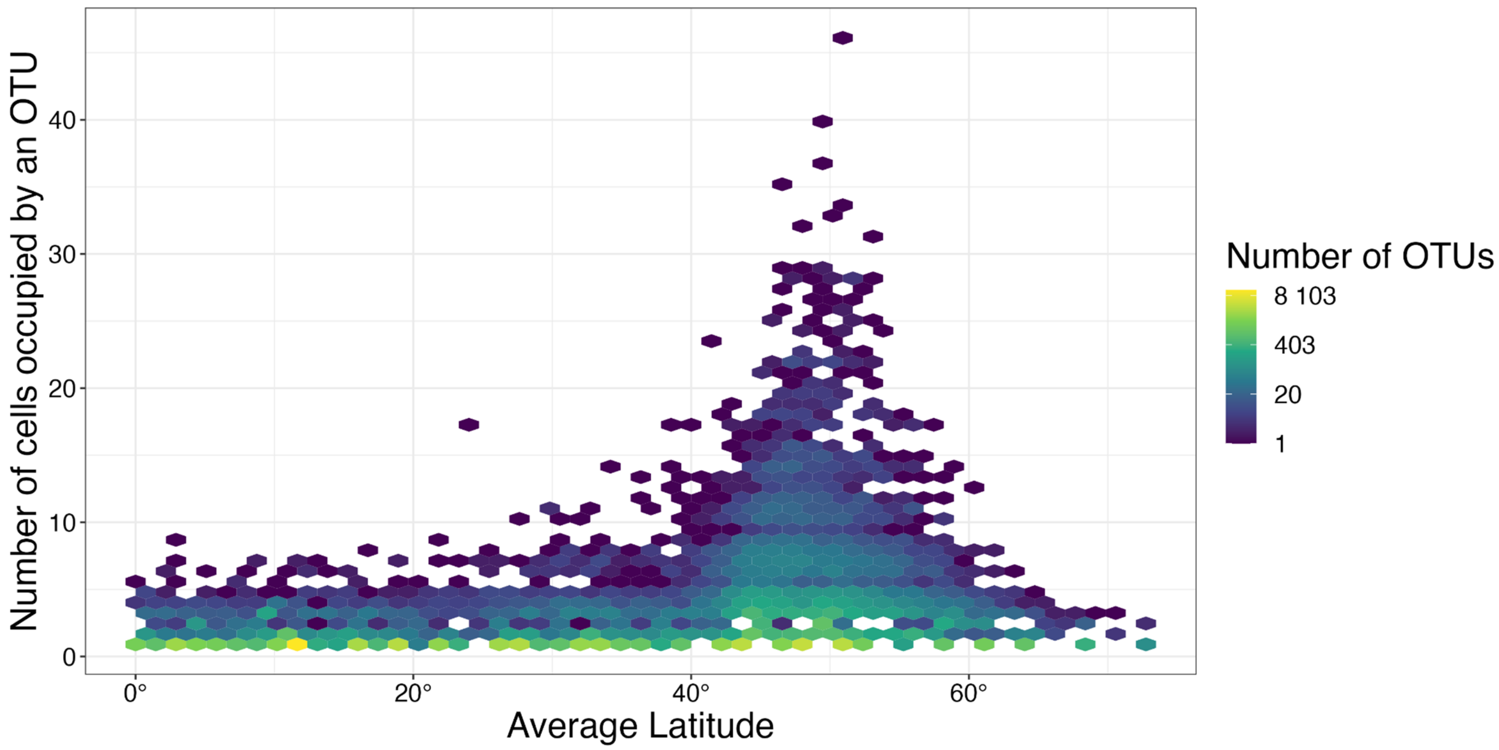

Supplementary Fig. 15

Simulations depicting the relationship between nucleotide diversity calculated for all sequences (*pi*) and for only unique haplotypes (*pi**) for 20 values of effective population size (*Ne*), equally spaced between 1e4 and 1e6. Figures show the average over 1000 simulations of the difference (left panel) or ratio (right panel) between *pi** and *pi* for three sampling regimes representing sample sizes of 5 (blue), 10 (orange), and 50 (green) samples per species. For both smaller *Ne* and larger sample sizes, *pi** is inflated with respect to *pi*, though the proportional size of this

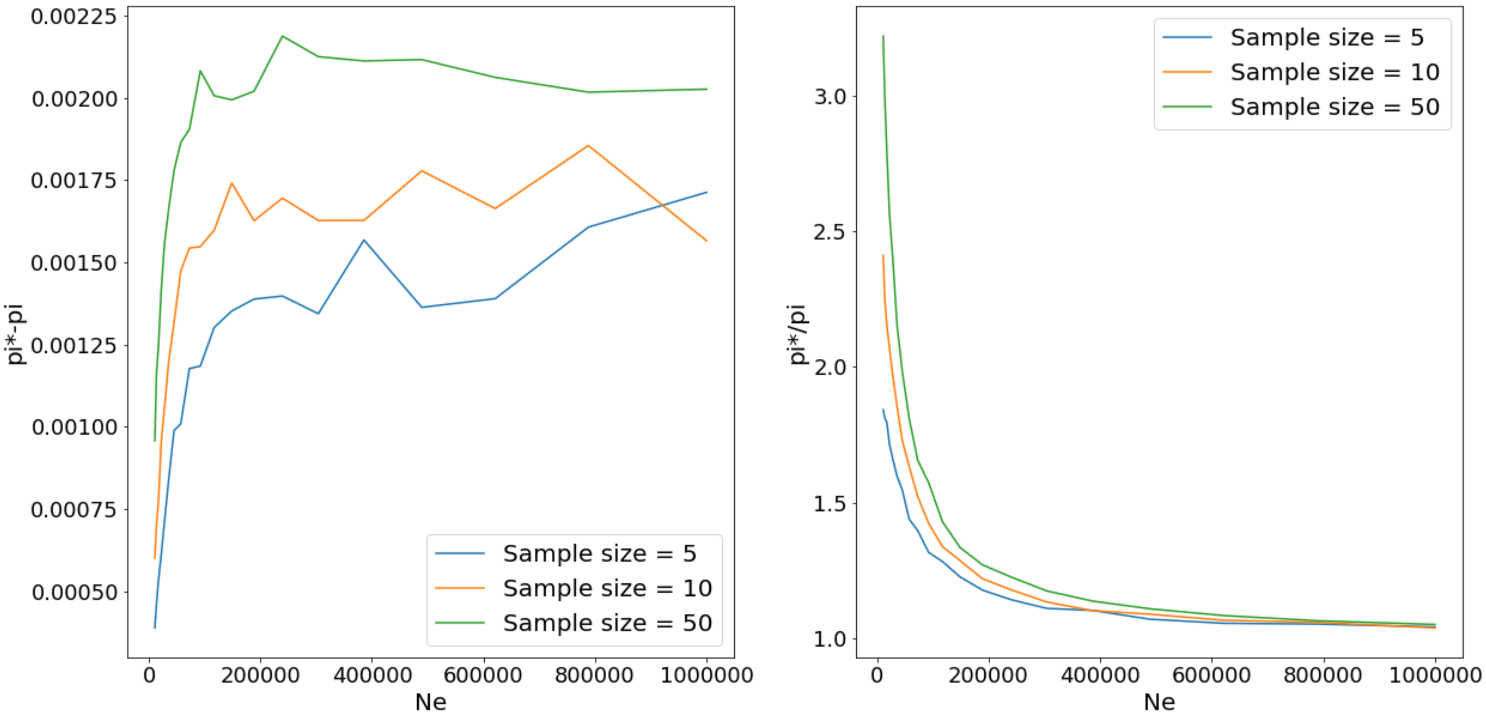

difference decreases with increasing *Ne*.

Supplementary Fig. 16

Scatterplots of genetic diversity mean (GDM) (a,b) and genetic diversity evenness (GDE) (c,d) and two measures of sampling effort, the number of individuals in a cell (a,c) and the number of operational taxonomic units (OTUs) in a cell (b,d) for the minimum OTU threshold of 100. The x-axes are log10-scaled for visualization purposes. No significant correlation exists between the sampling measures and either genetic diversity metric (see Supplementary Table 5).

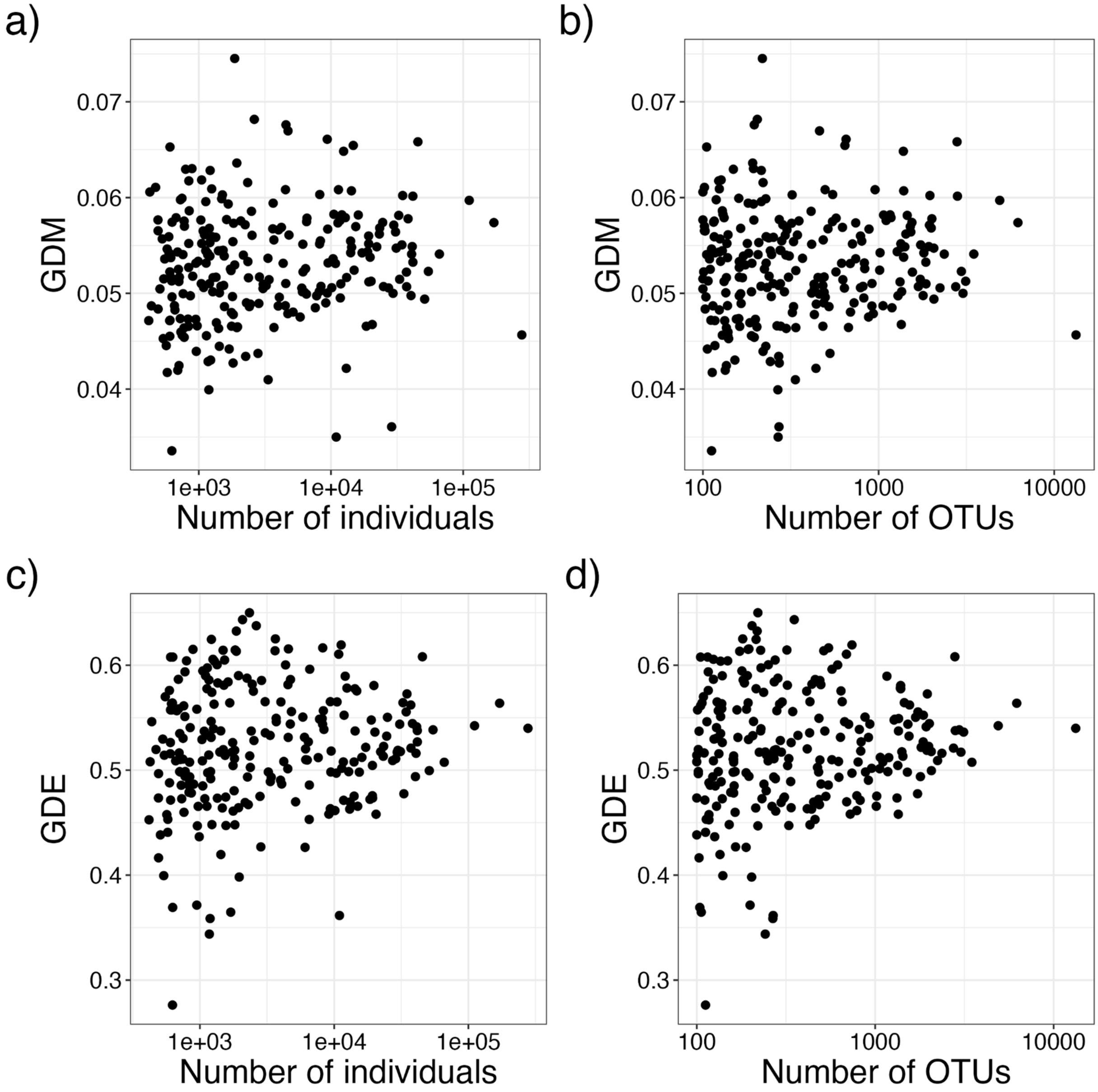
a)
b)
c)
d)
GDM
GDE
0.07
0.06
0.05
0.04
0.6
0.5
0.4
0.3
1e+03
1e+04
1e+05
100
1000
10000
Number of individuals
Number of OTUs

Supplementary Fig. 17

Sampling variability in estimates of genetic diversity mean (GDM) (a) and genetic diversity evenness (GDE) (b). We estimated potential sampling variability by resampling the ten assemblages with the largest number of operational taxonomic units (OTUs) (2,748 to 13,300 OTUs per grid cell) 1000 times without replacement, with 100 OTUs per sample. The vertical black lines indicate the genetic diversity value calculated with the full sample size.

a)

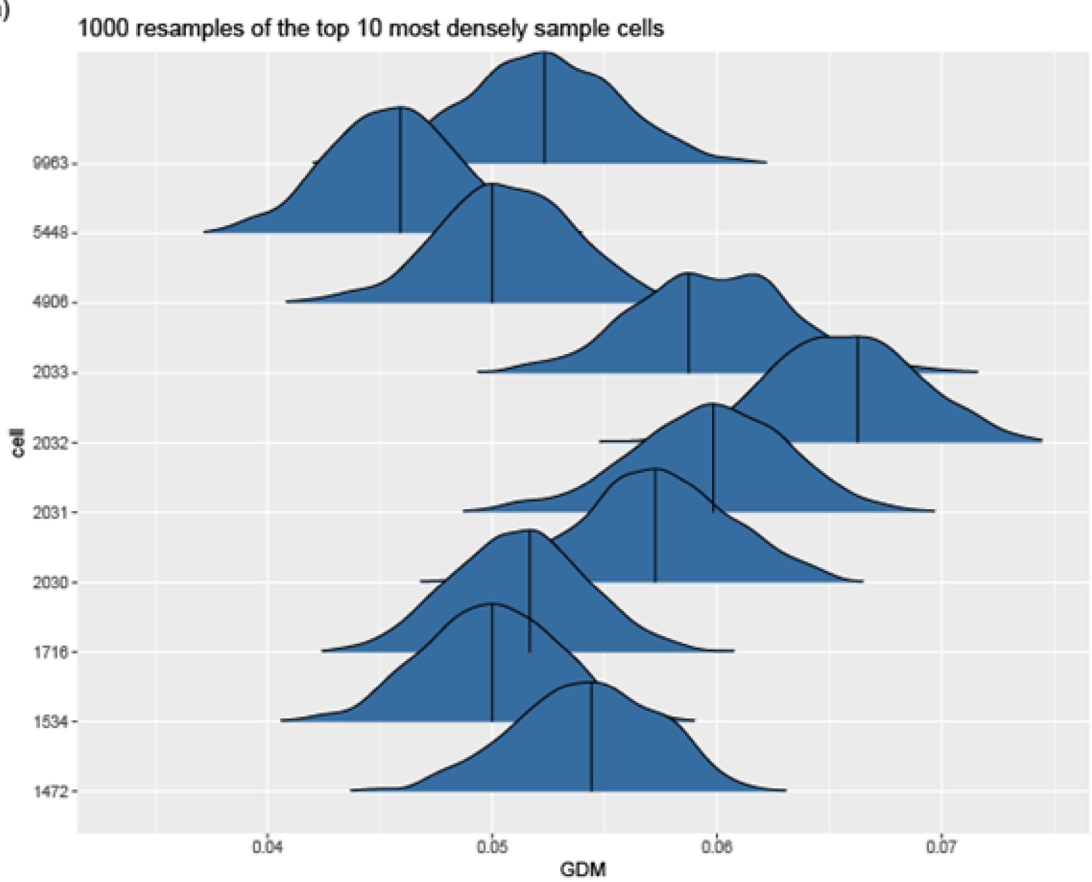
1000 resamples of the top 10 most densely sample cells
cell
9963
5448
4906
2033
2032
2031
2030
1716
1534
1472
0.04
0.05
0.06
0.07
GDM

b)

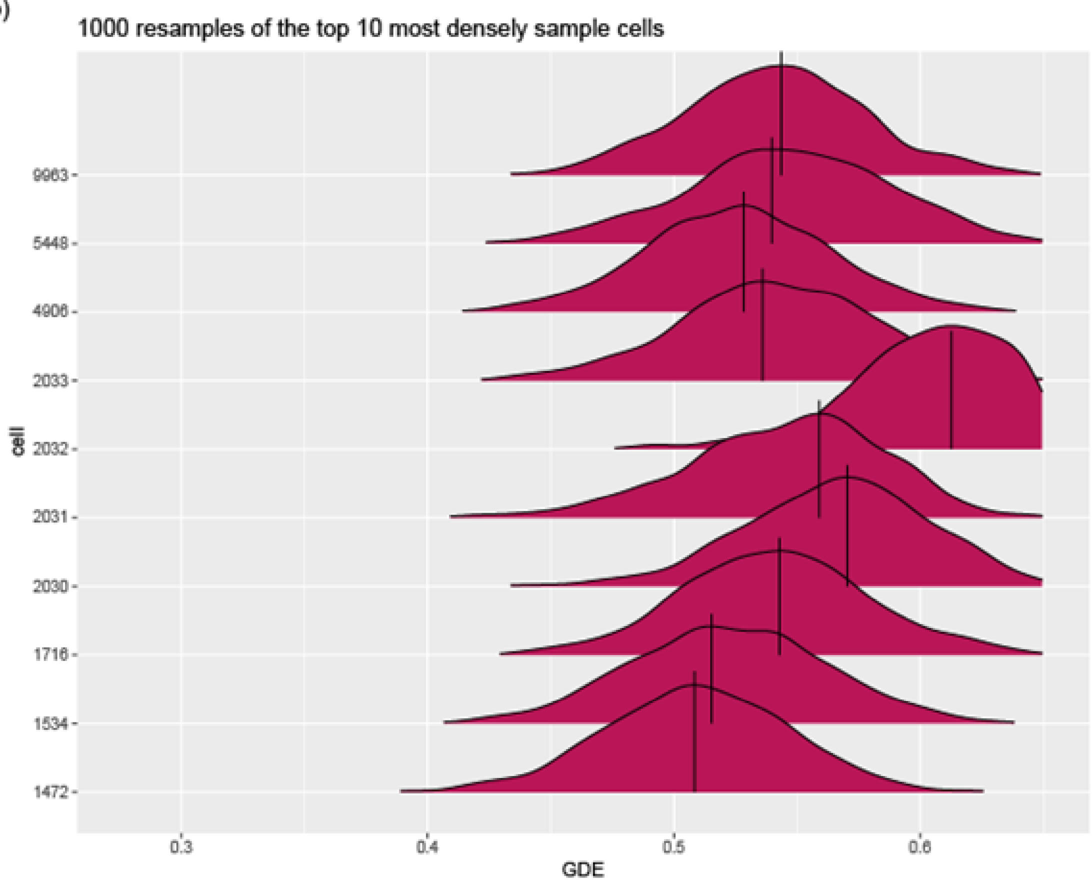
1000 resamples of the top 10 most densely sample cells
cell
9963
5448
4906
2033
2032
2031
2030
1716
1534
1472
0.3
0.4
0.5
0.6
GDE

Supplementary Table 1

Sampling details for all predictor variables considered for modeling.

Variable set	Resolution	Source	Number of variables	Time period	Citation
CHELSA Bioclims	10 arcmin	http://www.paleoclim.org/	19	1981-2010	1
Global Habitat Heterogeneity	12.5 arcmin	https://www.earthenv.org/texture	14	2001-2005	2
Elevation	2.5 arcmin	https://www.worldclim.org/data/worldclim21.html	2	NA	3
Slope	2.5 arcmin	https://www.worldclim.org/data/worldclim21.html	4	NA	3
Dynamic Habitat Indices	30 arcsecond	http://silvis.forest.wisc.edu/data/dhis/	3	2003-2014	4
Global Human Modification	30 arcsecond	https://figshare.com/articles/dataset/Global_Human_Modification/7283087	1	2016*	5
Climate stability	1 degree	https://github.com/spyrostheodoridis/Genetic-geography-of-terrestrial-mammals/blob/master/data.zip	4	21,000 BP - 1850 AD	6

*The time period for the Global Human Modification variable is reported as the median year used by the authors to derive the metric.

Supplementary Table 2

Summary statistics from projective prediction feature selection for genetic diversity mean (GDM).

Number of predictors	Predictors	ELPD	SE_{ELPD}	ELPD-DIF	$SE_{ELPD-DIF}$
0		677.8785	11.7596	-10.8082	6.0826
1	Precipitation trend	679.4131	14.5901	-9.2736	4.7335
2	Mean diurnal air temperature range	678.0864	14.2688	-10.6002	3.8210
3	Maximum temperature of the warmest month	680.4610	15.0807	-8.2257	3.9907
4	Precipitation seasonality	677.9266	15.1074	-10.7601	3.3189
5	Temperature trend	681.6466	15.0553	-7.0400	2.9160
6	Precipitation variation	684.5288	15.1132	-4.1579	2.7032
7	Precipitation of the wettest month	682.8683	14.9238	-5.8183	2.3163
8	Precipitation of the driest month	689.4619	14.1291	0.7753	1.2839
9	Temperature variation	687.9861	14.2599	-0.7005	1.0872
10	Global habitat heterogeneity (standard deviation)	687.4668	14.3596	-1.2199	1.0756
11	Human global habitat modification	687.2253	14.2297	-1.4614	0.8072

The ELPD is the expected log (pointwise) predictive density, where a higher ELPD indicates more support for the model. The predictor column is additive, where the model with the predictor listed contains all of the predictors listed before. In addition to ELPD, we report the difference in ELPD (ELPD-DIF), which is the difference in ELPD from the reference model. The $SE_{ELPD-DIF}$ is the standard error of the difference in ELPD from the reference model. SE_{ELPD} is a coarse summary of model support variation that is sensitive to sampling quality, and we take the advice of Vehtari et al. [7] to use ELPD-DIF and $SE_{ELPD-DIF}$ when comparing models. We chose the simplest model whose ELPD overlapped with the reference model's ELPD (ELPD-DIF + $SE_{ELPD-DIF}$ overlaps zero) and corroborated our selection with the heuristic method of selection employed by the *projpred* R package function *suggest_size()*. The chosen model is indicated with gray shading.

Supplementary Table 3

Summary statistics from projective prediction feature selection for genetic diversity evenness (GDE).

Number of variables	Predictors	ELPD	SE_{ELPD}	ELPD-DIF	$SE_{ELPD\text{-}DIF}$
0		253.652	11.704	-28.546	8.925
1	Maximum temperature of the warmest month	275.387	14.302	-6.812	4.981
2	Temperature trend	277.764	15.502	-4.435	4.165
3	Precipitation of the wettest month	279.314	17.078	-2.884	3.636
4	Temperature variation	285.854	16.165	3.655	2.258
5	Precipitation seasonality	286.469	16.720	4.270	1.815
6	Global habitat heterogeneity (standard deviation)	284.078	17.272	1.880	1.730
7	Precipitation variation	281.870	17.485	-0.329	1.698
8	Precipitation trend	280.279	18.175	-1.920	2.218
9	Precipitation of the driest month	280.526	17.899	-1.673	1.857
10	Mean diurnal air temperature range	279.379	17.846	-2.820	1.767
11	Human global habitat modification	280.342	17.869	-1.857	1.780

The ELPD is the expected log (pointwise) predictive density, where a higher ELPD indicates more support for the model. The predictor column is additive, where the model with the predictor listed contains all of the predictors listed before. In addition to ELPD, we report the difference in ELPD (ELPD-DIF), which is the difference in ELPD from the reference model. The $SE_{ELPD\text{-}DIF}$ is the standard error of the difference in ELPD from the reference model. SE_{ELPD} is a coarse summary of model support variation that is sensitive to sampling quality, and we take the advice of Vehtari et al. [7] to use ELPD-DIF and $SE_{ELPD\text{-}DIF}$ when comparing models. We chose the simplest model whose ELPD overlapped with the reference model's ELPD (ELPD-DIF + $SE_{ELPD\text{-}DIF}$ overlaps zero) and corroborated our selection with the heuristic method of selection employed by the *projpred* R package function *suggest_size()*. The chosen model is indicated with gray shading.

Supplementary Table 4

The raw number and proportion of operational taxonomic units (OTUs) belonging to each order in the dataset.

**Order**	**Number of OTUs**	**Proportion**
Diptera	64993	0.340
Lepidoptera	61914	0.324
Hymenoptera	33100	0.173
Coleoptera	14534	0.076
Hemiptera	7918	0.041
Trichoptera	2657	0.014
Ephemeroptera	1020	0.005
Orthoptera	918	0.005
Psocodea	811	0.004
Neuroptera	564	0.003
Odonata	608	0.003
Plecoptera	606	0.003
Thysanoptera	584	0.003
Blattodea	283	0.001
Mantodea	102	0.001
Archaeognatha	27	0.000
Dermaptera	20	0.000
Embioptera	13	0.000
Mecoptera	42	0.000
Megaloptera	61	0.000
Phasmatodea	20	0.000

Raphidioptera	10	0.000
Siphonaptera	33	0.000
Strepsiptera	7	0.000
Zygentoma	1	0.000
NA	46	0.000

Supplementary Table 5

Statistics from Pearson's correlation tests between genetic diversity (GD) metrics and three summaries of sampling effort.

**GD Metric**	**Predictor**	***r***	***t*-statistic**	**df**	***P*-value**
GDE	Total number of individuals per cell	0.064	1.001	243	0.318
GDM	Total number of individuals per cell	0.032	0.505	243	0.614
GDE	Median number of individuals per OTU	0.060	0.944	243	0.318
GDM	Median number of individuals per OTU	-0.030	-0.467	243	0.641
GDE	Number of OTUs per cell	0.080	1.243	243	0.212
GDM	Number of OTUs per cell	0.043	0.663	243	0.508

Supplementary Methods

Effect of duplicate alleles on genetic diversity

We used coalescent simulations in *msprime* [8] to explore the potential bias in calculation of nucleotide diversity (hereafter *pi*; [9]) where duplicate alleles may or may not be present in the data. The goal of this simulation study was to replicate data which may be obtained from online databases such as BOLD, where duplicate haplotypes within a focal species may not be represented by duplicate sequence records within the database, potentially biasing estimates of *pi*. For all simulations we set the sequence length to 500bp and the mutation rate to 1e-8/bp/generation, to approximate the features of a typical arthropod COI barcoding dataset. We explored combinations of three different sample sizes (*ss* = 5, 10, 50) to replicate shallow, moderate, and deep sampling of individuals, and a range of effective population sizes (*Ne*) for 20 values of *Ne* equally spaced between 1e4 and 1e6, thus capturing a wide range of *pi* values on the same order as those reported in the literature for arthropods [10]. We performed 1000 simulation replicates for each combination of sample size and *Ne*, calculated nucleotide diversity for all samples (*pi*) and for only those samples bearing unique haplotypes (*pi**), and recorded the value of the difference between (*pi** - *pi*) and the ratio between (*pi**/*pi*) these quantities averaged across all simulations.

Our coalescent simulation experiments showed a small but consistent upward bias in *pi** with respect to *pi* (Supplementary pi* Fig.). The average absolute difference between *pi** and *pi* increased with increasing *Ne* values uniformly for all sample sizes, while larger sample sizes showed consistently greater difference in *pi*-pi* across the spectrum of *Ne* values examined. In contrast, the relative difference between *pi** and *pi* decreased with increasing *Ne*, with *pi*/pi* approaching 1 as *Ne* approached 1e6 for all sample sizes. We draw three main conclusions from

these results: 1) The direction of the bias is consistent (*pi* > pi*) and predictable across a range of *Ne* values; 2) The bias is small for large *Ne* (> 1e6) which is typical for arthropods; 3) For small *Ne* (< 1e6), small sample sizes reduce both the absolute and relative difference between *pi** and *pi*. Taken together, and given the sample sizes and presumed *Ne* values of species from our empirical datasets, we conclude that the bias introduced by sampling only unique haplotypes should in most cases be small and predictable, and that, therefore, it should not unduly influence either the results or our main conclusions.

Modeling methods

We used projective prediction feature selection (see Methods) for variable selection [11]. This method projects the posterior information in the full model to smaller submodels by replacing the posterior distribution of the full model with a simpler distribution that is restricted by constraining relevant model parameters. In this case, the regression coefficients decline to zero. The predictive accuracy of these models was evaluated with an approximate leave-one-out (LOO) cross-validation technique, which avoids repeatedly fitting the full model by using an importance weighting scheme outlined in [7,11]. Under this framework, we used the expected log predictive density (ELPD), specifically the difference in ELPD from the reference model, as the utility function instead of other utility functions (*e.g.*, mean squared error) because it measures how well the predictive uncertainties are calibrated, in addition to measuring point predictions. While incorporating spatial autocorrelation into this variable selection step would be ideal, the full 11 variable set contains variables that do not necessarily vary linearly with the response and necessitates an exceedingly complex spatial covariance matrix, which results in the model failing to converge and displaying poor model diagnostics. Therefore, we opted for the simpler non-spatial approach to reduce the parameter space included in the final model.

Rather than estimating random effects at each location, which can be computationally intensive, a spatial field of correlated random effects at a subset of locations or "knots" was modeled. The choice of the number of knots is somewhat subjective, so we fit models using 5, 10, 15, 20, 25, and 30 knots. Models with 20 knots had the lowest residual SAC for GDE, while 30 knots had the lowest residual SAC for GDM, so we chose 20 knots for the GDE models and 30 knots for the GDM models. We used regularizing priors on all slope parameters (N(0, 0.1)) and sigma (N(0, 1)), as well as on the Gaussian process θ parameter (N(0, 5)), which controls how steeply the correlation between knots declines, and the Gaussian process sigma parameter (N(0, 1)), which controls the amplitude of spatial deviations. N() implies a normal distribution, where the first number in the parentheses is the mean and the second is the standard deviation.

Modeling output

Model output objects for genetic diversity mean (GDM) and genetic diversity evenness (GDE) at all minimum OTU thresholds (10, 25, 50, 100, 150, 200) are available at https://doi.org/10.6084/m9.figshare.c.6563836.v1. A guide for loading and interpreting the output is included in the repository and https://github.com/connor-french/global-insect-macrogenetics.

Correlation matrices

Correlation matrices of the proposed 49 variables are at https://doi.org/10.6084/m9.figshare.c.6563836.v1 as an Excel .xlsx workbook.

Supplementary References

1. Karger, D. N. *et al.* Climatologies at high resolution for the earth's land surface areas. *Scientific Data* **4**, 170122 (2017).
2. Tuanmu, M.-N. & Jetz, W. A global, remote sensing-based characterization of terrestrial habitat heterogeneity for biodiversity and ecosystem modelling. *Glob. Ecol. Biogeogr.* **24**, 1329–1339 (2015).
3. Fick, S. E. & Hijmans, R. J. WorldClim 2: new 1-km spatial resolution climate surfaces for global land areas. *Int. J. Climatol.* **37**, 4302–4315 (2017).
4. Hobi, M. L. *et al.* A comparison of Dynamic Habitat Indices derived from different MODIS products as predictors of avian species richness. *Remote Sens. Environ.* **195**, 142–152 (2017).
5. Kennedy, C. M., Oakleaf, J. R., Theobald, D. M., Baruch-Mordo, S. & Kiesecker, J. Managing the middle: A shift in conservation priorities based on the global human modification gradient. *Glob. Chang. Biol.* **25**, 811–826 (2019).
6. Theodoridis, S. *et al.* Evolutionary history and past climate change shape the distribution of genetic diversity in terrestrial mammals. *Nat. Commun.* **11**, 2557 (2020).
7. Vehtari, A., Gelman, A. & Gabry, J. Practical Bayesian model evaluation using leave-one-out cross-validation and WAIC. *Stat. Comput.* **27**, 1413–1432 (2017).
8. Baumdicker, F. *et al.* Efficient ancestry and mutation simulation with msprime 1.0. *Genetics* **220**, (2022).
9. Nei, M. & Li, W. H. Mathematical model for studying genetic variation in terms of restriction endonucleases. *Proc. Natl. Acad. Sci. U. S. A.* **76**, 5269–5273 (1979).

10. Leffler, E. M. *et al.* Revisiting an old riddle: what determines genetic diversity levels within species? *PLoS Biol.* **10**, e1001388 (2012).

11. Piironen, J., Paasiniemi, M. & Vehtari, A. Projective inference in high-dimensional problems: Prediction and feature selection. *EJSS* **14**, 2155–2197 (2020).
